# Laparoscopic lateral suspension combined with uterosacral ligament folding and shortening versus laparoscopic sacrocolpopexy for the treatment of pelvic organ prolapse: a retrospective cohort study

**DOI:** 10.3389/fmed.2025.1626735

**Published:** 2025-08-08

**Authors:** Xiangpeng Xiong, Leizhen Xia, Tong Mei, Jialv Huang, Ting Wang, Yun Zhang, Shuang Wang, Ye Luo, Haiping Liu, Liqun Wang, Xiaoyan Ai

**Affiliations:** ^1^Department of Gynecology, Jiangxi Maternal and Child Health Hospital, Nanchang, China; ^2^Center for Reproductive Medicine, Jiangxi Maternal and Child Health Hospital, Nanchang, China; ^3^Department of Ultrasound, Jiangxi Maternal and Child Health Hospital, Nanchang, China; ^4^Department of Imaging, Jiangxi Maternal and Child Health Hospital, Nanchang, China

**Keywords:** uterosacral ligament folding and shortening, laparoscopic sacrocolpopexy, pelvic organ prolapse, patient-reported outcomes, laparoscopic lateral suspension

## Abstract

**Background:**

Pelvic organ prolapse (POP) significantly impacts women's quality of life, with laparoscopic sacrocolpopexy (LSC) considered the gold standard for treatment. However, LSC carries risks of complications, prompting exploration of alternatives. This study compared the efficacy of Laparoscopic Lateral Suspension (LLS) combined with uterosacral ligament folding and shortening (LLS-ULFS) versus LSC for POP treatment.

**Overview:**

A retrospective cohort study included 445 women with POP-Q stage ≥ II (LSC group: n=232; LLS-ULFS group: n=213). Surgical outcomes, complications, and patient-reported outcomes were evaluated over a 2-year follow-up period at Jiangxi Maternal and Child Health Hospital.

**Results:**

Both groups achieved high anatomical success rates (LSC vs. LLS-ULFS): apical (96.98% vs. 94.84%), anterior (94.40% vs. 96.24%), and posterior (96.12% vs. 94.37%) compartments (all P>0.05). The LLS-ULFS group demonstrated superior perioperative outcomes: shorter operation time (85 vs. 105 min, P<0.001), reduced blood loss (40 vs. 50 ml, P<0.001), and lower pain scores (VAS: 4 vs. 4, P<0.001). Long-term follow-up showed significantly better patient-reported outcomes in the LLS-ULFS group for PFDI-20, POPDI-6, CRADI-8, and PISQ-12 scores (all P<0.05), indicating improved quality of life and sexual function. Complication rates were comparable (P>0.05).

**Discussion:**

LLS-ULFS achieves anatomical success equivalent to LSC while offering advantages in operative efficiency and recovery. The technique’s avoidance of presacral dissection likely contributes to reduced pain and complications.

**Conclusion:**

LLS-ULFS is a viable alternative to LSC, providing comparable anatomical correction with superior perioperative outcomes and enhanced quality of life. Its efficacy supports broader clinical adoption for POP management.

## 1 Introduction

Pelvic organs prolapse (POP) affects many women worldwide, causing a signifcant impact on quality of life (1), and the lifetime surgical risk is as high as 12.6% (2). The “three-compartment theory” categorizes the pelvic floor into three compartments: anterior (bladder, urethra, anterior vaginal wall), apical (uterus, vaginal vault), and posterior (posterior vaginal wall, rectum) (3). Studies have demonstrated that correction of apical pelvic prolapse can concurrently improve 50% of anterior compartment prolapse and 30% of posterior compartment prolapse (4). Currently, laparoscopic sacrocolpopexy (LSC) is regarded as the gold standard for treating POP, particularly in cases of severe apical or multi-compartment prolapse (5). However, LSC is a challenging procedure associated with rare but potentially severe complications, prompting clinicians to explore simpler and safer alternatives.

Laparoscopic lateral suspension (LLS) has emerged as a promising alternative to LSC (6). This technique involves passing sutures through the lateral peritoneal wall under the peritoneum, above the iliac crest, thereby reducing the risk of vascular, nerve, and bowel injuries (7). LLS is technically less complex compared to LSC, as it requires less dissection and less stitching and knot-tying, thus, making it more feasible via a minimally invasive approach. Research indicates that LLS is equally effective as LSC in correcting apical and anterior pelvic prolapse (8–10). However, for patients with multi-compartment prolapse, LLS falls short compared to LSC, particularly in addressing posterior compartment prolapse (11, 12). Continuous research and improvement are essential for enhancing the efficacy of any surgical technique. Studies have shown that uterosacral ligament folding and shortening can reorient the postoperative vaginal axis posteriorly, providing better support for the apical and posterior pelvic floor (13). In addition, based on the holistic theory, shortening and reinforcing the uterosacral ligament has been shown to alleviate symptoms of overactive bladder, nocturia, and urinary frequency in patients with POP (14). However, there is no research on the effect of LLS combined with sacral ligament folding and shortening surgery at present, whether the combination of the two surgical methods can improve the shortcomings of LLS in treating POP. Therefore, our objective was to achieve comprehensive treatment of POP through an individualized approach combining LLS with uterosacral ligament folding and shortening. This study aimed to compare surgical outcomes, complications, and prolapse-related symptoms between patients undergoing LSC and those undergoing LLS combined with uterosacral ligament folding and shortening.

## 2 Materials and methods

This retrospective cohort study was conducted at the Department of Gynecology, Jiangxi Maternal and Child Health Hospital. Patients with POP who underwent LSC or LLS combined with uterosacral ligament folding and shortening (LLS-ULFS) from September 1, 2017, to September 27, 2022, were screened for eligibility. Inclusion criteria were: (1) POP primarily involving apical pelvic prolapse (POP-Q stage ≥ II); (2) sexually active patients; (3) completed childbearing. Eligible patients included women of childbearing age and postmenopausal women. Concurrent surgeries such as hysterectomy, adnexectomy, stress urinary incontinence(SUI) surgery, or partial cervicectomy did not exclude patients from enrollment. Exclusion criteria included a history of previous pelvic floor surgery (e.g., transvaginal or abdominal autologous tissue or mesh surgery), severe medical comorbidities, coagulation disorders, acute inflammation, vaginal ulcers, severe pelvic adhesions, combined endometrial lesions, and inability to complete follow-up.

All surgeries were performed by gynecologists proficient in laparoscopic pelvic floor surgery. The study was approved by the Medical Ethics Committee of Jiangxi Maternal and Child Health Hospital (EC-KY-2024129), and informed consent was obtained from all patients for data use in the study.

### 2.1 Surgical methods

#### 2.1.1 Laparoscopic sacrocolpopexy group (LSC group)

**Figure 1:**
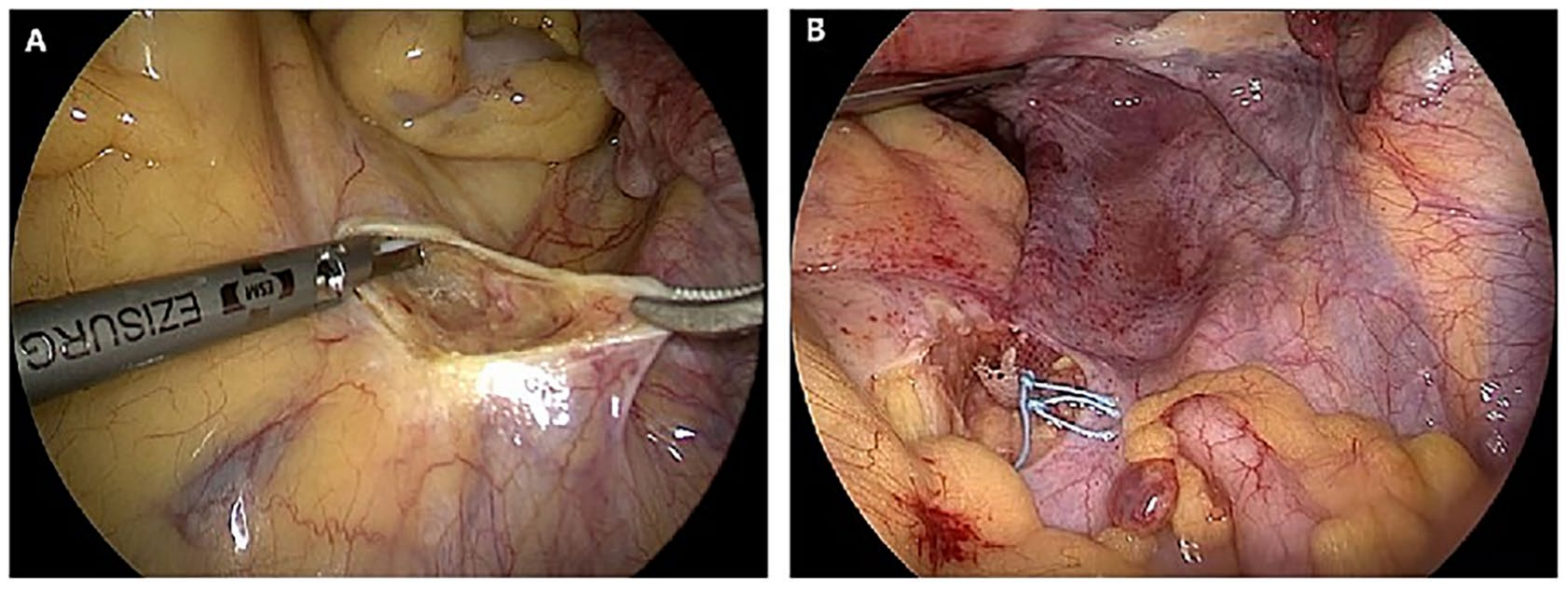
Laparoscopic sacrocolpopexy procedure. **(A)** Exposed the presacral region. **(B)** Secured the mesh to the presacral ligament.

After routine disinfection and draping, a 10 mm supra-umbilical incision was made to establish pneumoperitoneum. A laparoscope was inserted through the primary trocar. Additional 5 mm trocars were placed bilaterally at McBurney’s points and in the left mid-abdomen. The right paracolic gutter was exposed, and the right ureter was identified. The peritoneum anterior to the sacral promontory was longitudinally opened to expose the presacral space. The avascular area anterior to the S1 vertebral body was selected as the suture site. The lateral peritoneum was incised along the medial border of the right uterosacral ligament down to the vaginal fornix. If the uterus was present and indicated for removal, hysterectomy was performed first. For patients who opted for uterine preservation, the uterus was retained, and a hole was created in the right broad ligament to accommodate the mesh. An instrument for lifting the vaginal fornix was introduced vaginally to dissect the vesicovaginal and rectovaginal spaces. Hysterectomy was indicated for patients with uterine pathology (e.g., uterine tumors, endometriosis, adenomyosis, uterine prolapse), or patient preference. Uterine preservation was offered to those desiring fertility or without uterine pathology. The mesh was cut into a Y-shape and sutured to the vaginal apex and the anterior and posterior vaginal walls, respectively (if the uterus was retained, a hole was created in the right broad ligament to pull the vaginal mesh forward). The peritoneum anterior to the sacrum was opened to fully expose the anterior longitudinal ligament of the sacrum, and the mesh was secured to the presacral ligament. The peritoneal incisions were closed with absorbable sutures to ensure complete peritonealization (Figure 1).

#### 2.1.2 Laparoscopic lateral suspension combined with uterosacral ligament folding and shortening group (LLS-ULFS group)

**Figure 2:**
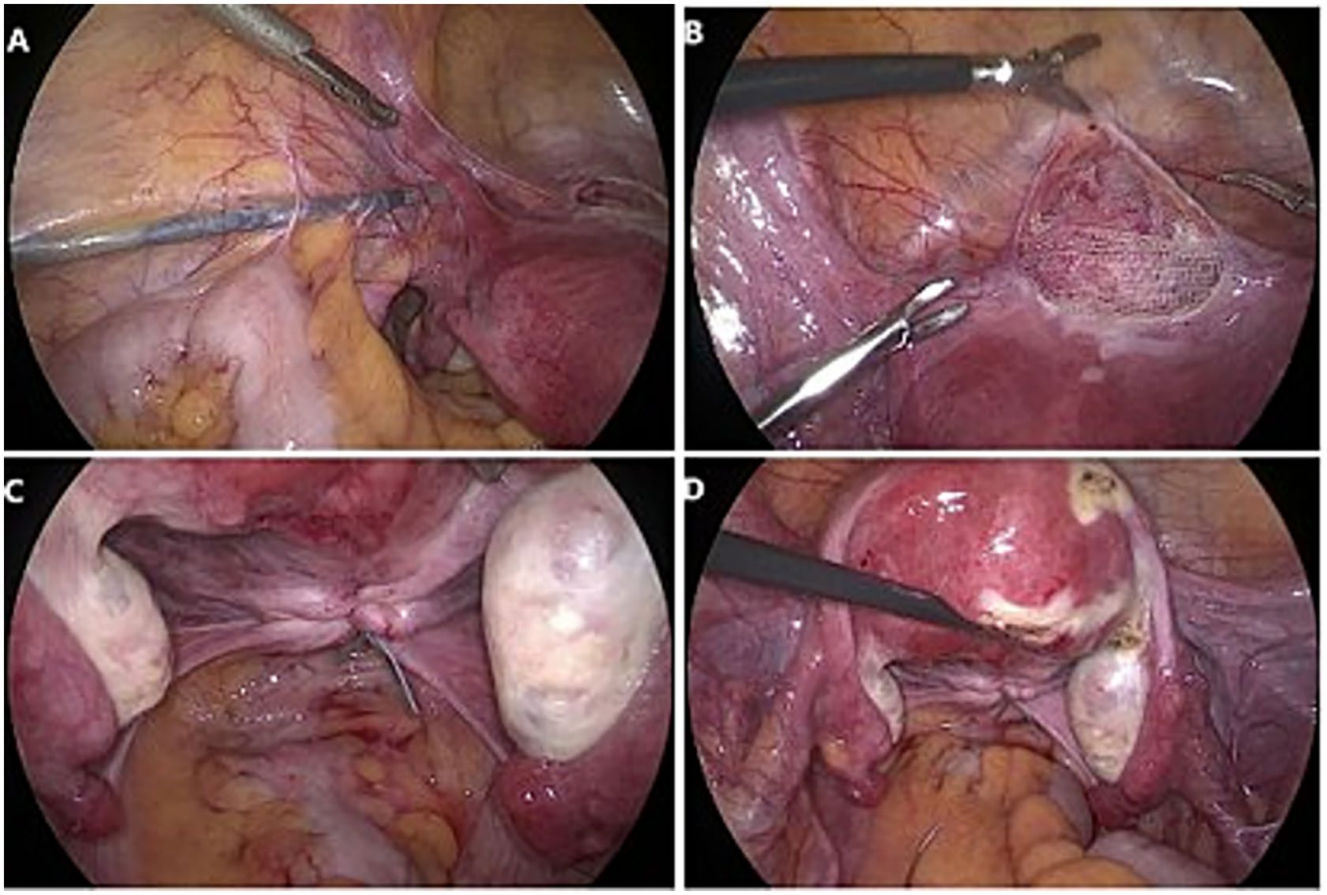
Laparoscopic lateral suspension combined with uterosacral ligament folding and shortening group procedure. **(A)** An extraperitoneal tunnel was established by maintaining a 40–45° angle with the round ligament. **(B)** The shorter arm of the mesh was centrally sutured and secured to the lower segment of the uterus and the anterior vaginal wall. **(C,D)** Uterosacral ligament folding and shortening, bilateral uterosacral ligaments were plicated, shortened, and sutured to the cervix using non-absorbable sutures.

After routine disinfection and draping, a 10 mm supra-umbilical incision was made to establish pneumoperitoneum. A laparoscope was inserted through the primary trocar. The second and third trocars were placed 4 cm above and 3 cm lateral to the anterior superior iliac spine, while the fourth trocar was positioned 5 cm below and to the left of the umbilicus. An instrument for lifting the vaginal fornix was introduced vaginally. The vesicouterine peritoneal reflection was fully dissected, creating a 5–6 cm space in the vesicovaginal region. A T-shaped mesh (with a short arm approximately 5 cm and a long arm approximately 15 cm) was introduced into the abdominal cavity. The dissector was utilized to traverse the abdominal fat and muscle layers, tunneling along the extraperitoneal space toward the round ligament. The tunnel was positioned beneath the round ligament at an angle of 40–45°relative to it. Grasping forceps were employed to extract the mesh arm through the tunnel to the exterior of the trocar. The mesh was then flattened, with the central portion of its short arm sutured and secured to the lower uterine segment and anterior vaginal wall (or to the vaginal stump and anterior vaginal wall if a hysterectomy had been performed). After adjusting the mesh tension, the peritoneum was continuously sutured to ensure complete peritoneal coverage (Figures 2A,B). The ureteral course was carefully examined, and the uterosacral ligaments were preserved. Bilateral uterosacral ligaments were plicated, shortened, and sutured to the cervix using non-absorbable sutures (or to the posterior vaginal stump in cases of hysterectomy) (Figures 2C,D). Excess mesh extending beyond the skin was excised, and the remaining mesh was sutured and embedded within the peritoneum using absorbable sutures.

If a hysterectomy was indicated, a total hysterectomy was performed concurrently; adnexal surgery was conducted if adnexal pathology was present; mid-urethral suspension was performed for moderate to severe stress urinary incontinence; and partial cervical resection was carried out if cervical elongation was noted. All surgeries utilized titanium-coated large-pore polypropylene mesh.

### 2.2 Outcome measurement and definition

Preoperative clinical data, including age, body mass index (BMI), obstetric history, surgical history, concurrent surgeries, symptoms, and anatomical evaluation (POP-Q staging, such as Ba, Bp, C) of all patients were meticulously recorded. The intraoperative conditions (operation duration, blood loss, length of stay, visual analog scale (VAS) score for pain, intraoperative and postoperative complications) were systematically evaluated. Clinical follow-up was conducted at 1 and 2 years post-surgery by experienced gynecologists. The primary outcome measure was the objective cure rate based on POP-Q stage. Prolapse stages across all sites were graded, and POP-Q scores were documented. Patients with a POP stage < II (all sites at least 1 cm above the hymen during Valsalva maneuver) were classified as anatomically successful. Quality of life and sexual satisfaction were assessed using standardized questionnaires before surgery and at 1 and 2 years post-surgery: Pelvic Organ Prolapse/Urinary Incontinence Sexual Function Questionnaire (PISQ-12) (the higher the score, the better the representative sexual function), and Pelvic Floor Impact Questionnaire-7 (PFIQ-7) (the higher the score is, the more serious the pelvic floor dysfunction problem will be). Pelvic Floor Distress Inventory (PFDI-20) (A high score indicates a more severe degree of prolapse), which includes the Pelvic Organ Prolapse Distress Inventory-6 (POPDI-6), Colorectal-Anal Distress Inventory-8 (CRADI-8) and Urinary Incontinence Distress Inventory-6 (UDI-6).

### 2.3 Statistical evaluation

Continuous variables were evaluated for normality via the Shapiro–Wilk test. Given that all variables deviated from a normal distribution, they were reported in the form of medians (25th percentile, 75th percentile) and then compared by means of the Mann–Whitney U test. Categorical variables, on the other hand, were presented as numbers and percentages and contrasted using the chi-square test, and if the sample size is less than 40 or the theoretical frequency is less than 5, Fisher’s exact probability method is used to test. To evaluate the isolated impacts of sacral ligament shortening, multiple logistic regression models were employed to obtain the odds ratios (ORs) for the binary clinical outcomes, while generalized estimation equation (GEE) models with linear regression were utilized to obtain the regression coefficient (B) for the non-normal continuous or graded clinical data. The model adjusted for various factors including all baseline data. Statistical analyses were performed using SAS version 9.4 (SAS Institute Inc., Cary, NC, Unites States). The significance level was set at two-tailed *p*-values < 0.05, indicating statistical significance.

## 3 Results

Hysterectomy was performed in 21.98% of LSC and 20.66% of LLS-ULFS patients. It is mainly used when there is an indication for surgical resection or the patient has a strong preference. A total of 445 patients met the eligibility criteria, comprising 232 patients who underwent LSC and 213 patients who underwent LLS-ULFS, all followed up for more than 2 years. Table 1 summarizes the demographic characteristics of the two groups. LSC Group had a significantly higher median age (58 years vs. 54 years, *p* = 0.004) and lower BMI (22.05 kg/m^2^ vs. 23.4 kg/m^2^, *p* = 0.001) compared to LLS-ULFS Group. There were no significant differences between the two groups in terms of the number of pregnancies, the proportion of prior cesarean section, Post-menopausal, prior surgery for the abdomen, and preoperative POP-Q stage. In terms of pelvic floor function and sexual function questionnaires, there was no significant difference in PFDI-20 scores between the two groups. However, the LSC group had a weaker impact of pelvic floor disorders with a significantly lower PFIQ-7 score (92.85 vs. 95.20, *p* < 0.001), and better sexual function with a significantly higher PISQ-12 score (20 vs. 19, *p* < 0.001) compared to the LLS-ULFS Group.

**Table 1:** Demographic features of patients in LSC group and LLS-ULFS group.

Characteristics	LSC (*n* = 232)	LLS-ULFS (*n* = 213)	*P*- value
Female age (years)	58.0 (51.0 ~ 61.5)	54.0 (50.0 ~ 60.0)	0.004
Female BMI (kg/m^2^)	22.05 (20.45 ~ 24.30)	23.40 (21.20 ~ 25.10)	0.001
Number of pregnancies	3 (2 ~ 4)	3 (2 ~ 5)	0.062
Prior cesarean section, *n* (%)	14 (6.03)	12 (5.63)	0.857
Post-menopausal, *n* (%)	92 (39.66)	93 (43.66)	0.392
Prior surgery for the abdomen, *n* (%)	32 (13.79)	27 (12.68)	0.729
POP-Q stage, *n* (%)			0.694
II	71 (30.60)	59 (27.70)	
III	149 (64.22)	140 (65.73)	
IV	12 (5.17)	14 (6.57)	
POP stage ≥ II, *n* (%)			
Anterior prolapse	232 (100)	207 (97.18)	0.012
Apical prolapse	225 (96.98)	212 (99.53)	0.070
Posterior prolapse	184 (79.31)	175 (82.16)	0.447
Anatomical points			
Ba	3 (1 ~ 4)	3 (1 ~ 5)	0.444
C	3 (1 ~ 5)	2 (1 ~ 4)	0.144
Bp	1 (−1 ~ 3)	1 (0 ~ 3)	0.550
Concomitant surgery, *n* (%)			
Hysterectomy	51 (21.98)	44 (20.66)	0.733
Adnexectomy	68 (29.31)	55 (25.82)	0.411
SUI surgery	32 (13.79)	24 (11.27)	0.422
Partial cervicectomy	45 (19.40)	39 (18.31)	0.770
Function score			
PFDI-20	100.00 (91.60 ~ 108.40)	100.00 (91.60 ~ 108.30)	0.486
POPDI-6	41.70 (37.50 ~ 45.80)	45.80 (37.50 ~ 50.00)	0.098
CRADI-8	16.70 (12.50 ~ 20.80)	16.70 (12.50 ~ 20.80)	0.000
UDI-6	41.70 (37.50 ~ 45.80)	37.50 (33.30 ~ 41.70)	<0.001
PFIQ-7	92.85(85.70 ~ 104.80)	95.20 (85.70 ~ 114.30)	<0.001
PISQ-12	20.00 (18.00 ~ 24.00)	19.00 (16.00 ~ 21.00)	<0.001

The surgical treatment conditions of the two groups are summarized in Table 2. LLS-ULFS group exhibited shorter operation duration (85 min vs. 105 min, *p* < 0.001), less intraoperative blood loss (40 mL vs. 50 mL, *p* < 0.001), and lower median postoperative VAS scores (4 vs. 4, *p* < 0.001) compared to LSC group. There was no significant difference in length of stay between the two groups (*p* = 0.190). Although LSC group experienced more perioperative and postoperative complications than LLS-ULFS group, this difference was not statistically significant (*p* > 0.05). In detail, the postoperative complications of the LSC group included 5 cases of mesh exposure, the LLS-ULFS group included 4 cases of mesh exposure, 7 cases of pelvic pain. Specifically, in LSC group, two patients developed postoperative hematoma which improved after conservative treatment. During postoperative follow-up, eight patients in LSC group reported low back pain, and one patient underwent discectomy due to sacral disc infection. At last, with further adjustment for demographic features, these results also maintained consistency.

**Table 2:** Surgical data of patients in LSC group and LLS-ULFS group.

Characteristics	LSC (*n* = 232)	LLS-ULFS (*n* = 213)	*P*-value	Parameter estimate (95% CI)	Standard error	*P*-value
Operation duration (min)	105 (95 ~ 125)	85 (70 ~ 110)	<0.001	0.17 (0.15 ~ 0.20)	0.01	<0.001
Blood loss (ml)	50 (40 ~ 60)	40 (30 ~ 55)	<0.001	0.17 (0.10 ~ 0.24)	0.04	<0.001
Length of stay (day)	4 (3 ~ 4)	4 (3 ~ 4)	0.190	0.04 (−0.02 ~ 0.10)	0.03	0.175
Median VAS score	4 (3 ~ 5)	4 (3 ~ 4)	<0.001	0.13 (0.08 ~ 0.19)	0.03	<0.001
Intraoperative complication, *n* (%)	10 (4.31)	7 (3.29)	0.574	0.12 (−0.52 ~ 0.75)	0.32	0.716
Postoperative complication, *n* (%)	33 (14.22)	21 (9.86)	0.159	0.19 (−0.18 ~ 0.56)	0.19	0.316

Parameter estimate was adjusted for all demographic features. For intraoperative complications and postoperative complications, parameter estimate refers to the odds ratio value, and for other indicators, parameter estimate refers to the regression coefficient. Recent complications: urinary tract injury, blood transfusion, severe bleeding / hematoma, infection, intestinal obstruction. Long-term complications: mesh exposure, pelvic pain, sacral back pain, new-onset stress urinary incontinence, new onset of overactive bladder, new onset of constipation, sexual dysfunction.

Recent complications occurred in 10 LSC patients (4.31%; 2 urinary tract injury, 1 blood transfusion, 2 severe bleeding / hematoma, 3 infection, 2 intestinal obstruction) and 7 LLS-ULFS patients (3.29%; 1 urinary tract injury, 3 blood transfusion, 1 severe bleeding / hematoma, 2 Infection). Long-term Complications, mesh exposure occurred in 5 LSC vs. 4 LLS-ULFS patients; pelvic pain in 2 LSC vs. 7 LLS-ULFS patients; sacral back pain affected 8 LSC patients; new-onset stress urinary incontinence in 7 LSC vs. 5 LLS-ULFS patients; new onset of overactive bladder in 4 LSC vs. 2 LLS-ULFS patients; a new onset of constipation in 4 LSC vs. 3 LLS-ULFS patients; sexual dysfunction in 3 LSC vs. 2 LLS-ULFS patients.

The postoperative follow-up conditions of the two groups are detailed in Tables 3, 4. Taking POP-Q stage < II indicates successful surgery, both groups showed significant improvement in anatomical conditions compared to preoperative levels. At 1 year post-surgery, the surgical success rates for anterior, apical, and posterior pelvic prolapse in LSC Group were 95.69, 97.41, and 96.55%, respectively, while those in LLS-ULFS Group were 97.18, 94.84, and 95.31%, respectively. At 2 years post-surgery, the surgical success rates for anterior, middle, and posterior pelvic prolapse in LSC Group were 94.40, 96.98, and 96.12%, respectively, while those in LLS-ULFS Group were 96.24, 94.84, and 94.37%, respectively. No significant differences were observed in surgical success rates between the two groups at 1 and 2 years post-surgery (*p* > 0.05).

**Table 3:** Postoperative first year situation for patients in LSC group and LLS-ULFS group.

Characteristics	LSC (*n* = 232)	LLS-ULFS (*n* = 213)	*P*-value	Parameter estimate (95% CI)	Standard error	*P*-value
POP stage < II, *n* (%)						
Anterior prolapse	222 (95.69)	207 (97.18)	0.767	0.21 (−0.43 ~ 0.85)	0.33	0.521
Apical prolapse	226 (97.41)	202(94.84)	0.156	−0.42 (−1.22 ~ 0.38)	0.41	0.308
Posterior prolapse	224 (96.55)	203(95.31)	0.505	−0.32 (−0.94 ~ 0.31)	0.32	0.318
Anatomical points						
Ba	−3 (−3 ~ −3)	−3 (−3 ~ −3)	0.848	0.02 (−0.12 ~ 0.16)	0.07	0.807
C	−7 (−7 ~ −6)	−7 (−7 ~ −6)	0.048	−0.21 (−0.53 ~ 0.12)	0.17	0.212
Bp	−3 (−3 ~ −3)	−3 (−3 ~ −3)	0.378	0.04 (−0.1 ~ 0.17)	0.07	0.587
Function score						
PFDI-20	18.7 (12.5 ~ 25)	16.6 (12.5 ~ 20.8)	<0.001	0.14 (0.06 ~ 0.23)	0.04	0.001
POPDI-6	4.2 (4.2 ~ 8.3)	4.2 (4.2 ~ 8.3)	0.088	0.2 (0.05 ~ 0.35)	0.08	0.009
CRADI-8	0 (0 ~ 4.2)	0 (0 ~ 4.2)	<0.001	0.92 (0.58 ~ 1.27)	0.18	<0.0001
UDI-6	8.3 (4.2 ~ 12.5)	8.3 (4.2 ~ 8.3)	0.054	−0.1 (−0.21 ~ 0.01)	0.06	0.085
PFIQ-7	9.5 (9.5 ~ 14.3)	9.5 (9.5 ~ 14.3)	0.081	0.02 (−0.13 ~ 0.17)	0.08	0.789
PISQ-12	30 (28 ~ 33)	30 (28 ~ 33)	0.214	−0.62 (−1.23 ~ −0.01)	0.31	0.045

Parameter estimate was adjusted for all demographic features. For POP stage, parameter estimate refers to the odds ratio value, and for other indicators, parameter estimate refers to the regression coefficient.

**Table 4:** Postoperative second year situation for patients in LSC group and LLS-ULFS group.

Characteristics	LSC (*n* = 232)	LLS-ULFS (*n* = 213)	*P*-value	Parameter estimate (95% CI)	Standard error	*P*-value
POP stage < II, *n* (%)						
Anterior prolapse	209 (94.40)	205 (96.24)	0.146	0.59 (−0.07 ~ 1.25)	0.34	0.080
Apical prolapse	225 (96.98)	202(94.84)	0.251	−0.35 (−1.07 ~ 0.38)	0.37	0.346
Posterior prolapse	223(96.12)	201 (94.37)	0.383	−0.3 (−0.85 ~ 0.25)	0.28	0.285
Anatomical points						
Ba	−3 (−3 ~ −3)	−3 (−3 ~ −3)	0.113	0.1 (−0.1 ~ 0.29)	0.10	0.329
C	−7 (−7 ~ −6)	−7 (−7 ~ −5)	0.024	−0.31 (−0.66 ~ 0.05)	0.18	0.087
Bp	−3 (−3 ~ −3)	−3 (−3 ~ −3)	0.922	−0.06 (−0.24 ~ 0.12)	0.09	0.505
Function score						
PFDI-20	20.8 (12.55 ~ 25)	16.6 (12.5 ~ 20.9)	<0.001	0.15 (0.07 ~ 0.23)	0.04	0.001
POPDI-6	8.3 (4.2 ~ 8.3)	4.2 (4.2 ~ 8.3)	0.065	0.17 (0.03 ~ 0.3)	0.07	0.014
CRADI-8	4.2 (0 ~ 8.3)	0 (0 ~ 4.2)	0.000	0.92 (0.6 ~ 1.25)	0.17	<0.0001
UDI-6	8.3 (4.2 ~ 12.5)	8.3 (4.2 ~ 12.5)	0.088	−0.09 (−0.2 ~ 0.02)	0.06	0.108
PFIQ-7	9.5 (9.5 ~ 14.3)	9.5 (9.5 ~ 19)	0.208	0 (−0.13 ~ 0.14)	0.07	0.949
PISQ-12	30 (28 ~ 32)	30 (28 ~ 33)	0.087	−2.07 (−2.54 ~ −1.6)	0.24	<0.0001

Parameter estimate was adjusted for all demographic features. For POP stage, parameter estimate refers to the odds ratio value, and for other indicators, parameter estimate refers to the regression coefficient.

Additionally, we recorded the conditions of Ba, Bp, and C points. At 1 and 2 years post-surgery, no significant differences were found between the two groups in Ba and Bp points (*p* > 0.05). Although initial observations suggested a difference in C point measurements, further regression analysis controlling for potential confounders revealed no significant difference between the two groups (B: −0.21, 95% CI: −0.53 ~ 0.12, *p* = 0.212; B: −0.31, 95% CI: −0.66 ~ 0.05, *p* = 0.087).

Furthermore, both groups showed significant improvements in PFDI-20, POPDI-6, CRADI-8, UDI-6, PFIQ-7 and PISQ-12 scores compared to pre-surgery levels. Notably, LLS-ULFS Group exhibited lower PFDI-20 scores than LSC Group at 1 and 2 years post-surgery (*p* = 0.001), indicating better quality of life. Similarly, LSC Group had significantly higher CRADI-8 scores than LLS-ULFS Group post-surgery (*p* < 0.0001). However, no significant differences were observed in POPDI-6, UDI-6, PFIQ-7, or PISQ-12 scores between the two groups post-surgery (*p* > 0.05). After further regression analysis, in addition to PFDI-20 and CRADI-8, it was also found that LLS-ULFS Group had better POPDI-6 and PISQ-12 scores during the 1 and 2-year follow-ups, with statistically significant differences (*p* < 0.05).

## 4 Discussion

The outcomes of our retrospective cohort study demonstrated that LLP-ULFS can achieve similar high anatomical cure rates for patients with POP compared to LSC. In addition, LLP-ULFS showed a better perioperative course of treatment, including shorter operation duration, less intraoperative blood loss, and less pain. What’s more, during long-term follow-up of 1–2 years, LLP-ULFS showed smaller effects of pelvic disorders as well as better sexual function.

LLS was first introduced by Dubuisson et al. (15) and represents the most recent alternative surgical technique for apical pelvic prolapse. In recent years, it has gained widespread clinical application. Multiple studies have demonstrated that, similar to LSC, LLS can achieve high anatomical cure rates for anterior and apical pelvic prolapse (10, 16–18). However, LLS falls short in addressing posterior pelvic prolapse, as the tension provided by the bilateral abdominal wall mesh cannot correct rectal uterine depression and may even exacerbate posterior pelvic prolapse (19). A study involving 417 patients with POP who underwent LLS reported a subjective cure rate of 78.4% at one-year follow-up, with anatomical cure rates of 91.6 and 93.6% for anterior and apical pelvic prolapse, respectively, but only 85.3% for posterior pelvic prolapse (20). This indicates that LLS is more suitable for patients with anterior and apical pelvic prolapse, while LSC is more effective for patients with anterior, apical, and posterior pelvic prolapse (21).

The primary objective of uterosacral ligament plication folding and shortening is to reinforce the support provided by the sacral ligaments. This procedure involves folding and suturing the bilateral sacral ligaments to the level of the cervix or vaginal stump using nonabsorbable sutures, thereby achieving sacral ligament shortening. This adjustment elevates the position of the uterus or vaginal stump, reorienting the postoperative vaginal axis posteriorly, which enhances the support of the apical and posterior pelvis and improves patients’ sexual function (22). To our knowledge, only one prior study has investigated the efficacy of LLS combined with sacroterine plication in treating apical prolapse. In a retrospective cohort study by Sahin et al. (23), 30 patients who underwent LLS surgery were compared with 30 patients who underwent LLS and sacroterine plication. The latter group demonstrated more significant improvements in sexual function and urinary symptoms, although there was no significant improvement in posterior compartments and anatomical Bp points compared to the LLS group (*p* = 0.312, *p* = 0.258). In our study, there was no significant difference in posterior compartments and anatomical Bp points between LSC group and LLS-ULFS group, and the anatomical cure rate was 96.1 and 94.4% at 2 years post-surgery, respectively (*p* > 0.05). These findings suggest that LLS combined with uterosacral ligament folding and shortening is comparable to LSC in treating anterior, apica and posterior pelvic prolapse, uterosacral ligament folding and shortening effectively compensates for LLS deficiencies in posterior compartment prolapse repair.

Based on the available evidence, LSC is a technically demanding procedure with rare but serious complications, including injury to major pelvic vessels, ureters, and the risk of sacral discitis (24, 25). This study found that the LSC group had 10 cases (4.31%) of perioperative complications and 33 cases (14.22%) of postoperative complications, both numerically higher than those in the LLS-ULFS group with no statistically significant difference. Specifically, 8 patients in the LSC group experienced severe low back pain due to sacral promontory fixation, which was difficult to relieve; one patient even required discectomy due to sacral disc infection. In contrast, no such complications were observed in the LLS-ULFS group. However, seven patients in the LLS-ULFS group reported pelvic pain, compared to only two patients in the LSC group. Additionally, despite the added time for uterosacral ligament folding and shortening, the overall operation time in the LLS-ULFS group was significantly shorter than that in the LSC group (85 min vs. 105 min, *P*<0.001). The VAS score for pain was also lower in the LLS combined with uterosacral ligament folding and shortening group (*p* < 0.001), and intraoperative blood loss was significantly reduced (*p* < 0.05). According to a systematic review (6), the mesh exposure rate for LLS ranges from 0 to 13%, similar to LSC (3 to 10%). However, our data showed lower mesh exposure rates in both groups, with only 5 cases in the LSC group and 4 cases in the LLS-ULFS group.

Our findings reveal that the LLS-ULFS group had significantly more favorable PFDI-20 scores than the LSC group, suggesting that LLS-ULFS causes less pelvic floor dysfunction disturbance. Our findings clearly demonstrate that the LLS-ULFS group achieved significantly more favorable PFDI-20 scores compared to the LSC group, strongly suggesting that the LLS-ULFS approach results in less disturbance from pelvic floor dysfunction. Moreover, the outcomes of the three subscales of PFDI-20 reveals that these improvements were predominantly observed in the outcomes of pelvic organ prolapse symptoms (POPDI-6), and bowel, rectal, anal symptoms (CRADI-8). In contrast, both procedures exhibited equivalent efficacy in terms of urinary tract symptoms (UDI-6). The underlying reasons for the superiority of LLS-ULFS are likely the avoidance of anatomical danger zones, lower surgical complexity, reduced trauma, and faster recovery (26). Dällenbach reported that 15 cases (27.8%) experienced preoperative constipation, with 11 cases (20.4%) still experiencing this condition postoperatively (27). In contrast, our approach of combining uterosacral ligament folding and shortening addressed rectal uterine depression and posterior pelvic prolapse, effectively alleviating constipation and other symptoms, with only 3 patients (1.4%) developing new-onset constipation postoperatively. Consequently, the CRADI-8 score was higher in our group compared to the LSC group. Our study also showed that LLS-ULFS had a better PISQ-12 score. The LLS procedure suspends the mesh horizontally along the uterine side wall, adhering to the original anatomical structure of the uterus, providing a more physiological vaginal axis orientation compared to LSC, this may explain the higher sexual satisfaction reported by patients in the LLS-ULFS (28).

To date, this is the first study to compare the efficacy of LLS combined with uterosacral ligament folding and shortening with LSC. Secondly, the content evaluated in this study is quite rich, including both the intraoperative and postoperative follow-up situations, as well as patient questionnaires. Lastly, the congruence between the multiple logistic regression model and the generalized estimation equation enhances the robustness and credibility of our findings.

There are several limitations to this study that should be acknowledged. First, retrospective cohort studies inherently carry potential biases and confounding factors. To mitigate patient recall bias, relevant clinical data were collected from identified medical records, and generalized linear regression analysis was conducted on the adjusted estimates. However, some confounders may still exist, such as variations in the gynecologists performing the POP procedures. Additionally, self-reported questionnaires on pelvic floor function may introduce certain deviations. Second, our study was conducted at a single center, which ensured consistency in clinical surgical techniques between the two groups but limited the robustness and generalizability of our conclusions. Finally, a 2-year follow-up period may not be sufficient to fully capture long-term postoperative complications and recurrence rates, warranting further monitoring in future studies.

In conclusion, our research demonstrates that for patients with POP, the LLS-ULFS approach can achieve a surgical success rate comparable to that of LSC. Moreover, the LLS-ULFS approach not only exhibits a more favorable perioperative treatment process but also significantly enhances the quality of life outcomes for patients. This implies that LLS-ULFS could potentially serve as an effective alternative treatment option in the management of POP. Even so, it will be necessary to ascertain our findings in well-designed and prospective studies.

## Data Availability

The raw data supporting the conclusions of this article will be made available by the authors, without undue reservation.

## References

[1] Weintraub AY Glinter H Marcus-Braun N. Narrative review of the epidemiology, diagnosis and pathophysiology of pelvic organ prolapse. Int Braz J Urol. (2020) 46:5–14. doi: 10.1590/s1677-5538.ibju.2018.0581, PMID: 31851453 PMC6968909

[2] Wu JM Matthews CA Conover MM Pate V Jonsson Funk M. Lifetime risk of stress urinary incontinence or pelvic organ prolapse surgery. Obstet Gynecol. (2014) 123:1201–6. doi: 10.1097/AOG.0000000000000286, PMID: 24807341 PMC4174312

[3] Maher C Yeung E Haya N Christmann-Schmid C Mowat A Chen Z. Surgery for women with apical vaginal prolapse. Cochrane Database Syst Rev. (2023) 7:CD12376. doi: 10.1002/14651858.CD012376.pub2, PMID: 37493538 PMC10370901

[4] Urogynecology Subgroup, Chinese Society of Obstetrics and Gynecology, Chinese Medical Association. Chinese guideline for the diagnosis and management of pelvic orang prolapse (2020 version). Zhonghua Fu Chan Ke Za Zhi. (2020) 55:300–6. doi: 10.3760/cma.j.cn112141-20200106-00016 32464716

[5] Maher CF Feiner B Decuyper EM Nichlos CJ Hickey KV O’Rourke P. Laparoscopic sacral colpopexy versus total vaginal mesh for vaginal vault prolapse: a randomized trial. Am J Obstet Gynecol. (2011) 204:360.e1–7. doi: 10.1016/j.ajog.2010.11.016 21306698

[6] Campagna G Vacca L Panico G Caramazza D Lombisani A Scambia G. Laparoscopic lateral suspension for pelvic organ prolapse: a systematic literature review. Eur J Obstet Gynecol Reprod Biol. (2021) 264:318–29. doi: 10.1016/j.ejogrb.2021.07.044, PMID: 34364019

[7] Dubuisson JB Veit-Rubin N Wenger JM Dubuisson J. Laparoscopic lateral suspension, another way to treat genital prolapse. Gynecol Obstet Fertil Senol. (2017) 45:32–6. doi: 10.1016/j.gofs.2016.12.009 28238313

[8] Szymczak P Grzybowska ME Wydra DG. Comparison of laparoscopic techniques for apical organ prolapse repair—a systematic review of the literature. Neurourol Urodyn. (2019) 38:2031–50. doi: 10.1002/nau.24115, PMID: 31452267

[9] Malanowska-Jarema E Starczewski A Melnyk M Oliveira D Balzarro M Rubillota E. A randomized clinical trial comparing Dubuisson laparoscopic lateral suspension with laparoscopic sacropexy for pelvic organ prolapse: short-term results. J Clin Med. (2024) 13. doi: 10.3390/jcm13051348, PMID: 38592190 PMC10931691

[10] Yassa M Tug N. Uterus-preserving laparoscopic lateral suspension with mesh operation in pelvic organ prolapse: initial experience in a single tertiary center with a median 24-month follow-up. Geburtshilfe Frauenheilkd. (2019) 79:983–92. doi: 10.1055/a-0941-3485, PMID: 31523099 PMC6739200

[11] Kumbasar S Salman S Sogut O K Gencer F Bacak HB Tezcan AD. Uterine-sparing laparoscopic lateral suspension in the treatment of pelvic organ prolapse. J Obstet Gynaecol Res. (2023) 49:341–9. doi: 10.1111/jog.15459, PMID: 36196844

[12] Tagliaferri V Taccaliti C Romano F D'Asta M Martulli B Gentile C. Laparoscopic sacrocolpopexy versus pelvic organ prolapse suspension for surgical management of pelvic organ prolapse: a retrospective study. J Obstet Gynaecol. (2022) 42:2075–81. doi: 10.1080/01443615.2021.2021508, PMID: 35129036

[13] Verma A Kashyap M Gupta A. High uterosacral ligament fixation versus McCall's culdoplasty for vaginal vault suspension in utero-vaginal prolapse surgery. Cureus. (2022) 14:e27368. doi: 10.7759/cureus.27368, PMID: 36046323 PMC9417864

[14] Liedl B Inoue H Sekiguchi Y Haverfield M Richardson P Yassourides A. Is overactive bladder in the female surgically curable by ligament repair? Cent European J Urol. (2017) 70:53–9. doi: 10.5173/ceju.2017.938, PMID: 28461989 PMC5407336

[15] Dubuisson J Yaron M Wenger J Dubuisson JB Wenger JM Jacob S. Treatment of genital prolapse by laparoscopic lateral suspension using mesh: a series of 73 patients. J Minim Invasive Gynecol. (2008) 15:49–55. doi: 10.1016/j.jmig.2007.11.003, PMID: 18262144

[16] Dubuisson J Eperon I Jacob S Dubuisson JB Wenger JM Dallenbach P. Laparoscopic repair of pelvic organ prolapse by lateral suspension with mesh: a continuous series of 218 patients. Gynecol Obstet Fertil. (2011) 39:127–31. doi: 10.1016/j.gyobfe.2010.12.007, PMID: 21377391

[17] Mereu L Tateo S D'Alterio MN Russo E Giannini A Mannella P. Laparoscopic lateral suspension with mesh for apical and anterior pelvic organ prolapse: a prospective double center study. Eur J Obstet Gynecol Reprod Biol. (2020) 244:16–20. doi: 10.1016/j.ejogrb.2019.10.026 31770687

[18] Isenlik BS Aksoy O Erol O Mulayim B. Comparison of laparoscopic lateral suspension and laparoscopic sacrocolpopexy with concurrent total laparoscopic hysterectomy for the treatment of pelvic organ prolapse: a randomized controlled clinical trial. Int Urogynecol J. (2023) 34:231–8. doi: 10.1007/s00192-022-05267-6, PMID: 35737006

[19] Simoncini T Russo E Mannella P Giannini A. Robotic-assisted apical lateral suspension for advanced pelvic organ prolapse: surgical technique and perioperative outcomes. Surg Endosc. (2016) 30:5647–55. doi: 10.1007/s00464-016-4924-8, PMID: 27287895

[20] Veit-Rubin N Dubuisson J Gayet-Ageron A Dubuisson JB Lange S Eperon I. Patient satisfaction after laparoscopic lateral suspension with mesh for pelvic organ prolapse: outcome report of a continuous series of 417 patients. Int Urogynecol J. (2017) 28:1685–93. doi: 10.1007/s00192-017-3327-2, PMID: 28417156

[21] Claerhout F De Ridder D Roovers JP Rommens H Spelzini F Vandenbroucke V. Medium-term anatomic and functional results of laparoscopic sacrocolpopexy beyond the learning curve. Eur Urol. (2009) 55:1459–68. doi: 10.1016/j.eururo.2008.12.008, PMID: 19111382

[22] Campagna G Panico G Lombisani A Vacca L Caramazza D Scambia G. Laparoscopic uterosacral ligament suspension: a comprehensive, systematic literature review. Eur J Obstet Gynecol Reprod Biol. (2022) 277:57–70. doi: 10.1016/j.ejogrb.2022.08.006, PMID: 36007356

[23] Sahin F Ozdemir S Dogan O. Should sacrouterine plication be added to lateral suspension surgery? A prospective study. J Obstet Gynaecol Res. (2024) 50:1042–50. doi: 10.1111/jog.15941 38627198

[24] Good MM Abele TA Balgobin S Schaffer JI Slocum P McIntire D. Preventing L5-S1 discitis associated with sacrocolpopexy. Obstet Gynecol. (2013) 121:285–90. doi: 10.1097/AOG.0b013e31827c61de, PMID: 23344278

[25] Veit-Rubin N Dubuisson J Lange S Dubuisson J-B Eperon I. Uterus-preserving laparoscopic lateral suspension with mesh for pelvic organ prolapse: a patient-centred outcome report and video of a continuous series of 245 patients. Int Urogynecol J. (2016) 27:491–3. doi: 10.1007/s00192-015-2859-6, PMID: 26476819

[26] Dubuisson JB Jacob S Chapron C Fauconnier A Decuypere F Dubernard G. Laparoscopic treatment of genital prolapse: lateral utero-vaginal suspension with 2 meshes. Results of a series of 47 patients. Gynecol Obstet Fertil. (2002) 30:114–20. doi: 10.1016/s1297-9589(01)00277-6, PMID: 11910879

[27] Dallenbach P Alec M Boulvain M Shabanov S. Outcomes of robotically assisted laparoscopic lateral suspension (RALLS) with mesh for anterior and apical prolapse. J Robot Surg. (2022) 16:287–94. doi: 10.1007/s11701-021-01234-3, PMID: 33821406 PMC8960596

[28] Pulatoglu C Yassa M Turan G Türkyılmaz D Doğan O. Vaginal axis on MRI after laparoscopic lateral mesh suspension surgery: a controlled study. Int Urogynecol J. (2021) 32:851–8. doi: 10.1007/s00192-020-04596-8, PMID: 33175232

